# An intervention for fear of progression in childhood cancer patients and their parents: feasibility and preliminary efficacy in a pilot randomised controlled trial

**DOI:** 10.1186/s12887-026-07201-x

**Published:** 2026-07-13

**Authors:** Jessy Herrmann, Laura Kern, Anja Santel, Kristina Herzog, Leonard Konstantin Kulisch, Christa Engelhardt-Lohrke, Rahel Hoffmann, Christiane Chen-Santel, Jörn-Sven Kühl, Florian Schepper

**Affiliations:** 1 Department of Paediatric Oncology Haematology & Haemostaseology, University Hospital Leipzig, Leipzig, Germany; 2 Elternhilfe für krebskranke Kinder Leipzig e.V (Parents’ Association for Children with Cancer Leipzig), Leipzig, Germany; 3 Department of Paediatric Oncology, University Hospital Carl Gustav Carus, Dresden, Germany; 4 Mental Health Research and Treatment Centre (FBZ), Faculty of Psychology, Ruhr-University Bochum and German Centre for Mental Health (DZPG), partner site Bochum/Marburg, Bochum, Germany; 5 Department of Paediatric Psychiatry, Psychotherapy, and Psychosomatics, University Hospital Leipzig, Leipzig, Germany

**Keywords:** Fear of Progression, Cancer, Paediatrics, Intervention, Feasibility

## Abstract

**Background:**

Fear of progression (FoP) is a significant psychosocial burden for children with cancer and their parents, influencing their quality of life (HRQoL) and emotional adjustment. In this pilot randomised controlled trial (RCT) we present results on feasibility and preliminary efficacy of the family-based therapeutic intervention “Kinder-Progredienzangst” (KIPA), targeting FoP in pediatric oncology.

**Methods:**

*N* = 29 families with a child undergoing acute cancer treatment or follow-up care completed the program, with participation open to parents alone or to parents and children; ultimately, *n* = 6 children participated. Eligibility criteria included moderate or high FoP in at least one family member. Families were randomised to either an intervention group or a waitlist-control group receiving treatment as usual. KIPA consists of psychoeducation, anxiety confrontation, and resource activation. Participants completed questionnaires for FoP, anxiety, depression, HRQoL, and posttraumatic stress symptoms (PTSS) at different time points. Participation and retention rates were calculated, and we used Mann-Whitney *U* tests for between-group comparisons, Friedman and Wilcoxon tests for within-group comparisons and Hedges *g* for effect sizes.

**Results:**

Feasibility results showed a participation rate of 23%, with higher participation in acute treatment (51%) compared to follow-up care (15%). Retention rates were 71% overall, with significant variability between settings. Efficacy analyses revealed significant differences in parental FoP between study conditions with high effect size (*W* = 65.5, *p*=.023, *g*=-0.855). Improvements were also noted in PTSS (*W* = 33, *p*<.001, *g*=-1.365), anxiety (*W* = 59, *p*=.017, *g*=-0.906) and mental (*W* = 217, *p*<.001, *g* = 1.619) and physical (*W* = 180, *p*=.042, *g* = 0.834) HRQoL. The pre-post analysis showed a significant reduction in parental FoP in both settings, and the follow-up data indicates sustainability. Effects on children’s FoP were not significant.

**Conclusion:**

The study showed mixed feasibility—particularly regarding child participation, follow-up enrolment and drop-out during acute treatment—highlighting the need for optimisation before larger trials. Nevertheless, the data provide initial indications that KIPA may benefit parents of children with cancer across both acute and follow-up care settings, a group highly affected by FoP. Given the methodological limitations, the efficacy results should be viewed as preliminary. To demonstrate KIPA’s effectiveness, especially in children, large multicentre trials using reliable RCT designs suitable for acute care settings are needed.

**Trial registration:**

The trial was retrospectively registered at the German Trial Registry (TRN: DRKS00024106, 04.05.2022).

**Supplementary Information:**

The online version contains supplementary material available at 10.1186/s12887-026-07201-x.

## Background

Fear of progression is defined as the fear that a severe, potentially life-threatening or incapacitating illness might progress or reoccur [1]. FoP is not dysfunctional per se, as it may facilitate compliance with medical procedures and favourable health behaviour [1]. Nevertheless, FoP (or the related construct fear of cancer recurrence FCR [2]), has been reported as a major psychosocial burden paediatric cancer patients and their parents [3, 4].

Knowledge about the level of burden of FoP in paediatric oncology is scarce, and the comparability of study results is limited due to different cut-offs. Studies suggest that 48.3–75.1% of parents of childhood cancer patients experience elevated FoP [5–7] These high prevalence rates may be caused by feelings of helplessness and loss of control in the role of a responsible caregiver as well as a lack of self-care [7]. Children have been shown to be less burdened by FoP, although studies providing comparable information on the prevalence of elevated FoP in children are lacking [4, 8, 9].

Findings on medical (e.g., time since diagnosis) and sociodemographic (e.g., age) variables associated with elevated FoP are inconsistent [3, 6, 7, 9]. Decreased health-related quality of life (HRQoL) in paediatric patients and parents [6, 10] as well as psychological distress, such as anxiety, depression and posttraumatic stress symptoms (PTSS; [4–7]), have been shown to be associated with elevated FoP.

Theoretical models emphasize that FoP is a nearly ubiquitous experience among patients and family members, triggered or maintained by uncertainty (e.g., limited information about recurrence risk), individual vulnerability factors (e.g., previous loss experiences), and maladaptive metacognitive beliefs (e.g., the meaning attributed to recurrence-related worries) [11]. Based on these models, persistent cognitive engagement with worries as well as attempts to avoid such thoughts are assumed to play a central role. Consequently, existing interventions in adult oncology aim to promote a more functional way of responding to distressing cognitions and the strong emotions associated with them [12].

Regarding FCR in children and adolescents, a framework highlights cognitive (e.g., understanding of the illness) and social (e.g., parent–child relationship) factors that shape FCR in childhood and emphasizes the central role of parental behaviours [13]. This conceptualization positions FCR in paediatric oncology as a family-level phenomenon, which is also supported by empirical evidence [14, 15] and suggests that family-oriented interventions are indicated, as implemented in other psychosocial domains [16].

Although interventions addressing FoP in adult cancer patients [17] and their family caregivers [18] have been presented, evidence-based interventions targeting FoP in children and/or their parents are still in their infancy and, to the best of our knowledge, only one protocol of a paediatric FoP intervention has been published [19], with no pilot or efficacy results reported to date. Therefore, we considered the development of family-oriented interventions in paediatric oncology [20–23] and piloted the intervention “Kinder-Progredienzangst” (KIPA). Initial results show that participating children, parents and professionals indicate a high level of satisfaction [24]. The professionals also rated their adherence to the manual as high and considered the applicability of the intervention with different age groups, family dyads and settings to be satisfactory [24]. The aim of this study is to provide data on the feasibility and preliminary efficacy of KIPA to equipe future definitive RCT´s in different treatment phases:

- (RQ1) To evaluate the feasibility of KIPA, we analyzed (a) participation and (b) retention.
- (RQ2) To evaluate the effects of KIPA, we examined

- (a) differences in FoP and
- (b) secondary outcomes between the intervention group and waitlist controls,
- (c) changes in children´s FoP and parental FoP before and after KIPA and 4 months later,
- (d) differences by treatment status and
- (e) other sociodemographic variables

## Methods

### Design, participants and procedure

**Fig. 1:**
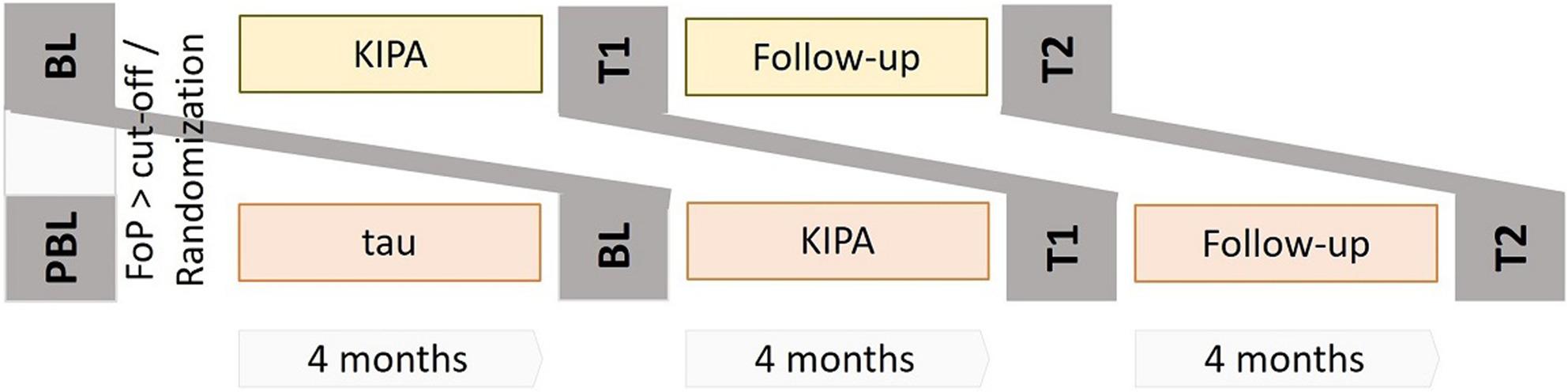
Design of the KIPA study. Note: BL: Baseline, PBL: Pre-Baseline, T1: Timepoint after intervention, T2: Timepoint after follow-up, tau: Treatment as usual, KIPA: Kinder-Progredienzangst

Figure 1 shows the waiting list randomised controlled design. The participants in the intervention group had three measurement points: before (BL) and after (T1) the intervention and four months after (T2). Waitlist controls had an additional measurement point before their 4-month waiting period (prebaseline, PBL). The CONSORT Statement extension to randomised pilot and feasibility trials [25] was applied in this study.

Families were recruited on the paediatric oncology ward at the University Hospital Leipzig and in an outpatient cancer counselling centre in Leipzig (Elternhilfe für krebskranke Kinder Leipzig e.V., Germany). On the acute ward, families were approached by the psychosocial care team when they met general inclusion criteria and elevated FoP was noted during the routine initial psychosocial assessment. In the follow-up setting, all clients who had received counselling within the past ten years and met the general inclusion criteria were contacted by email and invited to participate. In both settings, formal FoP levels required for determining eligibility were assessed only after families had provided written informed consent.

The inclusion criteria were (1) patients aged 0–17 years with a new or relapsed cancer diagnosis of any entity within the last ten years and a minimum time of four weeks since diagnosis and their families and (2) minimum moderate FoP level of at least one family member applying a two-stage cut-off for parents (FoP-Q-SF/*P* ≥ 26; [3]) and children (FoP-Q-SF/C ≥ 19; [4]). The exclusion criteria were (1) insufficient German language skills, (2) inability to follow the intervention due to cognitive impairments assessed by the treating psychosocial staff, and (3) palliative care.

After providing written informed consent, parents and/or children completed one-time questionnaires on sociodemographic characteristics as well as FoP and secondary outcomes in paper-pencil form at each measurement time point. Families were–separately for acute treatment and follow-up care–randomly assigned to either the intervention group or the waitlist-control group receiving usual care [26]. To maintain balanced group sizes, we applied a restricted randomisation procedure using computer generated fixed-sized blocks with a predetermined equal distribution of intervention and control assignments [27]. All allocation sequences were generated prior to recruitment and remained fully concealed from the psychosocial case workers at the study centres who enrolled participants. Study coordinator JH assigned each family to the next allocation in the pre-generated sequences.

**Fig. 2:**
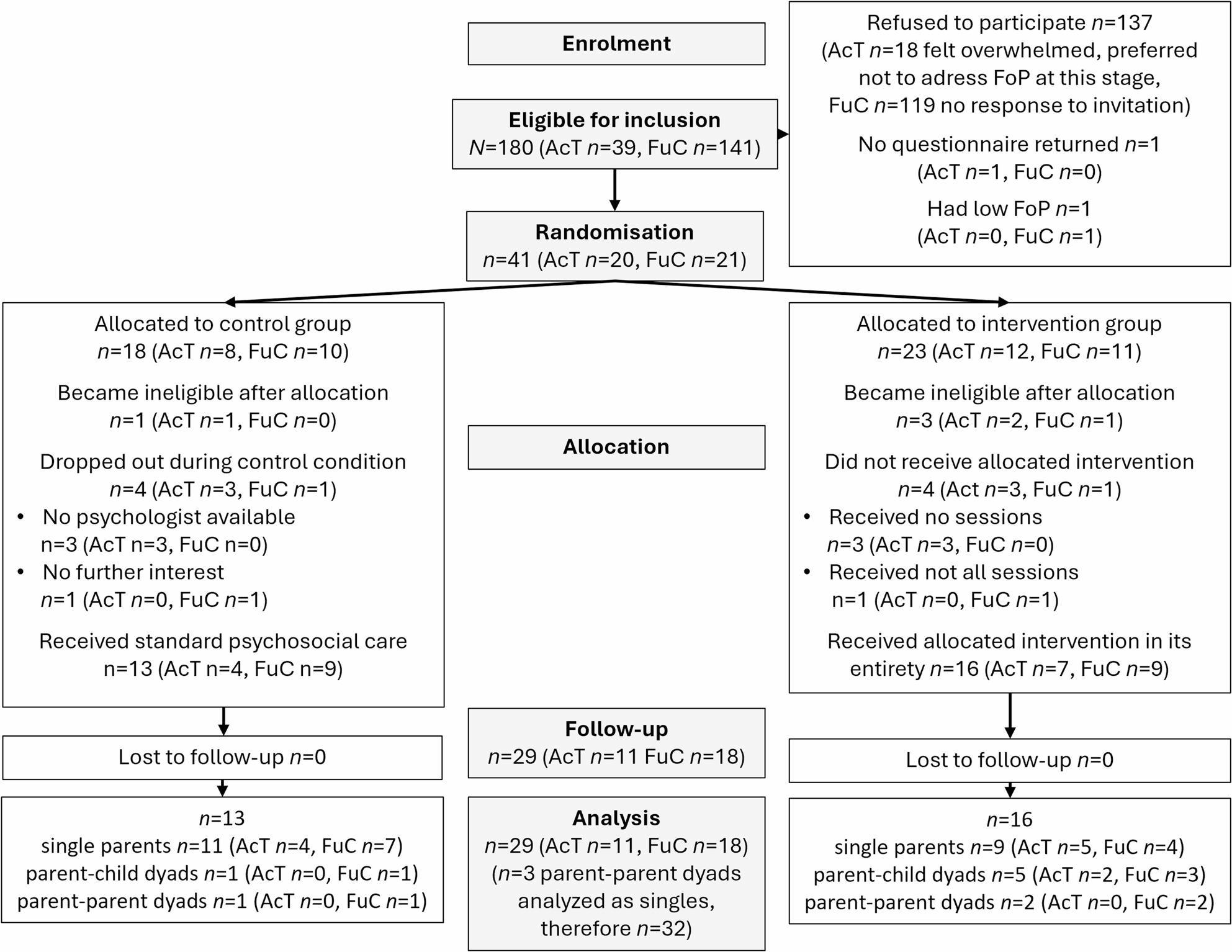
Flow chart for participation and drop-out

As the flow chart for participation and dropout (Fig. 2) shows, 180 families were eligible for inclusion and informed about the study; *n* = 41 were randomised, and *n* = 29 finished the study. As this was a pilot trial, participation was open to parents alone or parent-child dyads, upon request we as well allowed the participation of parent-parent dyads. Furthermore, we had two families in follow-up care who received large parts of the intervention online due to COVID-19 restrictions.

To determine the sample size required to assess the feasibility and effects of KIPA and design future RCTs accordingly, we followed Whitehead et al. [28] and aimed for two arms, each with *n* = 15 participants, assuming a medium effect size of KIPA. The planned recruitment period (08/2021–10/2022) was extended by nine months due to high drop-out rates on the acute ward, which resulted from COVID-19–related staffing shortages. These circumstances also made it increasingly difficult to deliver the waitlist-control condition as intended. Therefore, in April 2023, after an adequate number of control participants had been enrolled, randomisation was suspended and all subsequently recruited families on the acute ward were allocated to the intervention group to ensure the continued feasibility of the study. Recruitment concluded once the sample size was sufficient for the planned analyses. Additionally, a focus group was conducted to explore psychosocial professionals’ perspectives on the feasibility of implementing KIPA in both settings [24].

Due to COVID-19 related staffing shortages and resulting attrition, the planned intention-to-treat analysis was not feasible. Therefore, we conducted a per-protocol analysis including only families who completed the required assessments. To assess potential bias related to missing data, we additionally performed a drop-out analysis comparing baseline characteristics between completers and non-completers. *N* = 29 families with a child with cancer were included in efficacy analysis. One parent (*n* = 20), a parent‒child (older than six years) dyad (*n* = 6) or both parents (analysed as singles, *n* = 6) participated. In total, *N* = 32 data sets were analysed, including *n* = 14 participants in the control group and *n* = 18 in the intervention group. Sociodemographic and medical characteristics are shown in Table 1.

**Table 1:** Sociodemographic and illness characteristics of the sample

Children	Intervention group (*n* = 16)	Control group (*n* = 13)	All (*n* = 29)
Age in years M (SD)	8.5 (5.5)	7.9 (3.8)	8.3 (4.7)
Gender n (%)			
male	11 (69%)	8 (62%)	19 (66%)
female	5 (31%)	5 (39%)	10 (35%)
Diagnosis n (%)			
Leukaemia	2 (13%)	8 (53%)	10 (35%)
Tumour of the central nervous system	3 (19%)	1 (7%)	4 (14%)
Lymphoma	1 (6%)	-	1 (3%)
Tumours of peripheral nerves	-	1 (7%)	1 (3%)
Soft-tissue sarkoma	3 (19%)	-	3 (10%)
Kidney Tumour	3 (19%)	2 (13%)	5 (17%)
Bone Tumour	-	1 (7%)	1 (3%)
Other (e.g. dysgerminoma)	4 (25%)	-	4 (14%)
Time since diagnosis in years M (SD)	2.0 (2.3)	3.0 (2.9)	2.4 (2.6)
Treatment n (%) - mulitiple responses possible			
Chemotherapy	13 (81%)	12 (80%)	25 (86%)
Radiotherapy	3 (19%)	2 (13%)	5 (17%)
Surgical measures	10 (63%)	8 (53%)	18 (62%)
Bone narrow/stem cell transplant	-	1 (7%)	1 (3%)
Other (e.g. immune therapy)	2 (13%)	1 (7%)	3 (10%)

Participating parents	*n* = 18	*n* = 14	*n* = 32
Age in years M (SD)	37.9 (5.2)	40.4 (6.5)	39 (5.9)
Gender n (%)			
male	3 (17%)	2 (14%)	5 (16%)
female	15 (83%)	12 (86%)	27 (84%)

Participating children	*n* = 5	*n* = 1	*n* = 6
Age in years M (SD)	12.5 (2.7)	17.4	13.3 (3.2)
Gender n (%)			
male	3 (60%)	1 (100%)	4 (67%)
female	2 (40%)	-	2 (33%)
Diagnosis n (%)			
Leukaemia	-	-	-
Tumour of the central nervous system	1 (20%)	1 (100%)	2 (33%)
Lymphoma	1 (20%)	-	1 (17%)
Tumours of peripheral nerves	-	-	-
Soft-tissue sarkoma	1 (20%)	-	1 (17%)
Kidney Tumour	1 (20%)	-	1 (17%)
Bone Tumour	-	-	-
Other (e.g. dysgerminoma)	1 (20%)	-	1 (17%)
Time since diagnosis in years M (SD)	1.9 (2.7)		1.9 (2.7)
Treatment n (%) - mulitiple responses possible			
Chemotherapy	4 (80%)	-	4 (67%)
Radiotherapy	-	1 (100%)	1 (17%)
Surgical measures	3 (60%)	1 (100%)	4 (67%)
Bone narrow/stem cell transplant	-	-	-
Other (e.g. immune therapy)	1 (20%)	-	1 (17%)

*IG* intervention group, *CG* control group, *M* mean, *SD* standard deviation

### Measures

#### Fear of progression questionnaire short-form for children with cancer and their parents (FoP-Q-SF/C and P)

Children and parents indicate their FoP at each measurement point on twelve items on a five-point Likert scale ranging from (1) *never* to (5) *very often*. The total score ranges from 12 to 60, with higher scores indicating higher levels of FoP. The FoP-Q-SF/P for parents of children with cancer shows good reliability (Cronbach´s *α* = 0.89) and validity (7). This also applies to the adaptation of the questionnaire for children with cancer (Cronbach´s *α* = 0.87 [29]).

To date, no established cut-off values for dysfunctional fear of progression (FoP) are available, and several alternative thresholds have been proposed [9]. Based on our clinical experience indicating that patients with moderate to high FoP typically require psychosocial support, we applied an empirical two-stage cut-off—previously suggested by Luz et al. [4] and Schepper et al. [3]—to determine eligibility (i.e., moderate and high FoP) for study participation.

#### Secondary outcomes

We assessed parental anxiety and depression with the German version of the Hospital Anxiety and Depression Scale (HADS; [30]), HRQoL was measured with the Short Form-36 Health Survey [31], and posttraumatic stress symptoms were measured with the German version of the PCL-5 [32]. To assess children’s anxiety, we used subscales from the German version of the Fear Survey Schedule for Children–Revised (PHOKI; [33]). We measured depressive symptoms with the DTK-II short form [34], HRQoL with the KIDSCREEN-10 [35] and posttraumatic stress symptoms with the CATS-D for children and adolescents [36].

### Intervention

KIPA is based on cognitive behavioural and solution-focused therapeutic approaches and was developed in consideration of a recent theory of FoP, therapeutic manuals to treat FoP in adult oncology patients [12, 37] and programs addressing fear and depression in children [38, 39].

See Appendix 1 (Appendix, docx) for details of the intervention and Schepper et al. [40] for the therapeutic material (comic, pictures).

KIPA is designed so that family dyads can participate together or that a parent or child can participate individually, e.g., if the child cannot participate due to its young age (0–6 years) or low FoP, but the parent shows elevated FoP. In this case, a special focus is placed on attachment and the child’s perspective using circular conversation techniques in psychoeducation and training.

The intervention was carried out by psychosocial case workers (psychologists, social workers) from the participating institutions, usually with one-to-three-week intervals between sessions.

### Statistical analysis

Analyses were performed via R version 4.2.2. The significance level was set to *α* ≤ 0.05 for all analyses.

To examine feasibility, the percentage of families who were enrolled (RQ1a) and who withdrew (RQ1b) is reported, as well as differences in withdrawal across study conditions. Furthermore, we explored whether retention was related to demographic factors (age, sex), disease factors (diagnosis, time since diagnosis) and scores on baseline psychosocial measures by comparing parents who withdrew and those who remained using chi-square and Mann-Whitney-*U*-tests.

To analyse group differences in the main and secondary outcomes (RQ2a), we first ensured that randomisation yielded comparable groups and analysed psychosocial status by study condition at baseline. We had to control for between-group differences before the intervention/waiting period (BL/PBL). Despite randomisation, such differences may occur in small samples and mask correlations after the intervention/waiting period (T1/BL). We used regression analyses to control for variable levels via residualisation. After *z*-standardization and proof of normal distribution (Shapiro‒Wilk test), we used the Mann-Whitney-*U*-test to estimate group differences as well as Hedge’s *g* effect sizes after the intervention/waiting period (T1/BL). Additionally, we calculated differences in FoP within the waitlist control group from PBL to BL via the Wilcoxon test. Owing to the small number of participating children, we ran this analysis only for parents.

To evaluate the pre-post and long-term intervention effects (RQ2c), we calculated the differences in children’s and parents’ FoP between three measurement points (BL, T1, T2) via the Friedman test and Kendall´s ω as well as the Wilcoxon test and Hedge’s *g* effect size for the post hoc test [41]. We further explored efficacy across settings (RQ2d) through pre-post comparisons (BL to T1) of parental FoP using the Wilcoxon test and Hedges’ g and examined associations with sociodemographic variables (RQ2e) age of the child, perceived level of financial security and parental education using linear regression.

We evaluated the statistical power post hoc via g*power.

## Results

### Feasibility (RQ 1)

Overall participation (RQ 1a) was 23%, and retention (RQ 1b) was 71%, with substantial differences between acute treatment and follow-up care.

In acute treatment, psychosocial case workers invited *n* = 39 eligible families with assumed elevated FoP; *n* = 21 agreed to participate. Refusals were due to feeling too overwhelmed and not wanting to address their FoP at that stage. All interested families met the FoP threshold, and *n* = 1 did not return the questionnaire. *N* = 20 families were randomised (participation rate 51%), and *n* = 11 families completed the study (retention rate 55%). Reasons for drop-out included becoming ineligible after allocation due to a child´s death (*n* = 3) and withdrawing before the intervention began (*n* = 6). Among these six, *n* = 1 withdrew due to family burden, *n* = 1 left the acute ward and for *n* = 4 families no psychologist was available in time because of high patient volumes (*n* = 1 in the intervention group and *n* = 3 in the waitlist-control group).

In follow-up care, *n* = 141 eligible clients (initial consultation for psychosocial support within the last 10 years) were contacted by mail; *n* = 22 families wished to participate, and *n* = 1 had low FoP. Thus, *n* = 21 families were randomised (participation 15%), and *n* = 18 completed the study (retention 86%). Drop-outs occurred due to becoming ineligible after allocation (*n* = 1), losing interest before the start of intervention (*n* = 1), and declining participation after a change of psychosocial case workers (*n* = 1).

There were no significant differences in withdrawal between the KIPA (*n* = 7) and TAU (*n* = 5, *p*=.781) study conditions. The families who withdrew did not differ significantly from study completers in terms of, age (*W* = 135.5, *p*=.1422) or diagnosis (*χ²*_*[7]*_ = 4.22, *p*=.754) of the child, age (*W* = 184, *p*=.344) and gender (*χ²*_*[1]*_ = 2.61, *p*=.106) of the parents, time since diagnosis (*W* = 70, *p*=.215), or parental scores on FoP (*W* = 130.5, *p*=.946), anxiety (*W* = 138.5, *p*=.625), PTSS (*W* = 168, *p*=.181), and mental (*W* = 109, *p*=.532) and physical (*W* = 117.5, *p*=.734) subscales of HRQoL. Regarding depression (*W* = 174, *p*=.019) were the dropouts significantly more burdened.

### Efficacy: between-group differences in FoP (RQ 2 A) and secondary outcomes (RQ 2B) for parents

Randomisation produced comparable groups (intervention *n* = 18, waitlist control *n* = 14), and baseline equivalence was confirmed, with all pre-intervention differences small and non-significant. As shown in Table 2, the intervention group reported significantly lower FoP, anxiety, and PTSS, as well as higher mental and physical HRQoL at post-assessment. These effects were accompanied by moderate to very large effect sizes. 

**Table 2:** Group comparisons between intervention and control group

Outcome	Test statistic	*p*-value	Hedges g [95% CI]
FoP (Baseline)	W = 165	0.143	0.54 [-0.18, 1.26]
Anxiety (Baseline)	W = 142	0.555	0.25 [-0.46, 0.96]
Depression (Baseline)	W = 105	0.643	-0.10 [-0.82, 0.63]
PTSS (Baseline)	W = 142.5	0.543	0.24 [-0.47, 0.95]
HRQoL – Mental (Baseline)	W = 121.5	0.879	-0.24 [-0.96, 0.47]
HRQoL – Physical (Baseline)	W = 148	0.412	0.40 [-0.32, 1.12]
FoP (Post)	W = 65.5	0.023	-0.86 [-1.60, -0.12]
Depression (Post)	W = 65.5	0.146	-0.58 [-1.35, 0.20]
Anxiety (Post)	W = 59	0.017	-0.91 [-1.66, -0.15]
PTSS (Post)	W = 33	< 0.001	-1.37 [-2.15, -0.58]
HRQoL – Mental (Post)	W = 217	< 0.001	1.62 [0.80, 2.44]
HRQoL – Physical (Post)	W = 180	0.042	0.83 [0.10, 1.57]

*W* Wilcoxon rank-sum test statistic. Effect sizes are reported as Hedges g with 95% confidence intervals. Negative g values indicate higher symptom burden in the control group; positive values indicate higher scores in the intervention group (for HRQoL outcomes)

Additional analysis of the course of parental FoP within the waitlist control group from PBL to BL (waiting period) revealed a nonsignificant result (*W =* 54, *p=.*950*)*.

### Pre-post and long-term intervention effects (RQ 2 C)

**Fig. 3:**
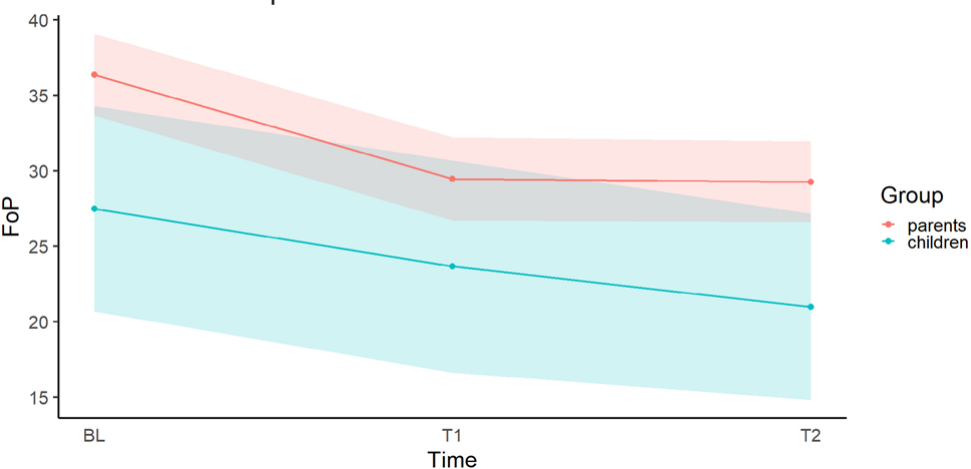
Course of FoP (M, 95% CI) in parents and children

To investigate RQ 2c the full sample (*n* = 32) was analysed. Figure 3 shows the course of parents’ and children’s FoP before (BL) and after (T1) the intervention as well as at follow-up (T2).

Parental FoP differed moderately across the three assessments (BL, T1, T2) (χ²_[2]_ = 24.14, *p*<.001, ω = 0.377, *CI* [0.18;0.62]). Post hoc tests revealed a significant difference in parental FoP before (BL) and after (T1) the intervention, with a large effect size (*W* = 462.5, *p*<.001, *g* = 0.855; 95% *CI* [0.517;1.193]), as well as before (BL) and four months after the intervention (T2) (*W* = 503, *p*<.001, *g* = 0.893; 95% *CI* [0.586; 1.218]).

Concerning children´s FoP, there was a small difference among the three assessments (χ²_[2]_ = 7.91, *p*=.019, ω = 0.659, 95% *CI* [0.58;0.94]). However, a post hoc test revealed no meaningful differences in children´s FoP between BL and T1 (*W* = 15, *p*=.438, *g* = 0.373, 95% CI [-0,460; 1.206]) or between BL and T2 (*W* = 15, *p*=.059, *g* = 0.671, 95% CI [-0,231; 1.573]).

### Pre- and postintervention effects by treatment status (RQ 2D)

Pre-post analysis of parental FoP separately by treatment status (RQ 2d, *n* = 26) revealed a significant difference between BL and T1 for parents in acute treatment (*W* = 55, *p*=.006, *g* = 0.747; 95% *CI* [0.402;1.093]) and follow-up care (*W* = 208, *p*=.001, *g* = 0.861; 95% *CI* [0.387;1.335]).

### Associations with sociodemographic variables (RQ 2E)

A multiple linear regression predicting FoP scores at T1 from baseline FoP, child’s age, perceived financial security, and educational level showed that baseline FoP was a highly significant predictor, whereas none of the other variables contributed significantly to the model. Detailed results are reported in Appendix 2 (Appendix, docx).

### Post hoc power analysis

The post hoc power analysis for the Mann–Whitney U test examining group differences in parental FoP (RQ 2a) yielded a power of 1–β = 0.740 (*n* = 32, g = –0.855, α = 0.05).

Wilcoxon tests assessing parental FoP across the three assessments (RQ 2c) showed a power of 1–β = 0.99 for both BL–T1 (g = 0.855) and BL–T2 (g = 0.893) comparisons (*n* = 32, α = 0.05).

For children’s FoP (*n* = 6, α = 0.05), the power was 1–β = 0.191 for BL–T1 (g = 0.373) and 1–β = 0.394 for BL–T2 (g = 0.671).

Wilcoxon tests comparing parental FoP between BL and T1 in the acute-treatment and follow-up groups (RQ 2d) showed a power of 1–β = 0.725 for parents in acute treatment (g = 0.747, *n* = 11) and 1–β = 0.981 for those in follow-up care (g = 0.861, *n* = 21).

## Discussion

### Feasibility

This study addressed a notable gap in the literature, as no previous intervention on FoP in paediatric oncology has been systematically evaluated, making the assessment of feasibility an essential first step.

To draw conclusions on the feasibility of KIPA, overall low participation (RQ 1a, 23%) must be differentiated between settings in which the study was conducted. Participation in acute treatment (51%) was good when considering that studies have shown recruiting parents of newly diagnosed children undergoing acute cancer treatment can be very difficult [21–23]. Facilitating factors in the acute treatment setting of this study included the individualized approach by psychosocial professionals and the minimal organizational effort required from families to participate during inpatient treatment [20]. In contrast, participation in follow-up care (15%) was low, as families were contacted by mail up to 10 years after diagnosis. Time constraints, geographical distances and the absence of childcare options [42] could have been further barriers and the manualisation of online formats could further promote participation in follow-up care [43]. The greater psychosocial burden on parents during acute treatment [5, 44], particularly the greater FoP burden [3, 6] than during follow-up care, could have impacted their motivation to participate.

A study retention rate of 71% was acceptable when considering previous findings [21, 22, 45], although substantial differences emerged between the acute treatment setting (55%) and follow-up care (86%). The long waiting time in the control group, unpredictable disease progression and very volatile patient volume in the acute ward led to dropout, especially during acute treatment, which reduces interpretability of the efficacy results. A dropout analysis revealed that they did not differ significantly from completers in terms of sociodemographic and medical factors, except for their higher level of depression. This finding is consistent with studies indicating that a higher baseline severity of depression predicts higher dropout rates [46]. Apart from this and because there was low dropout after the intervention started, KIPA appears to be feasible in both settings, which is also emphasized by psychosocial professionals [24].

A more detailed examination of feasibility for children is warranted, given their very low participation (*n* = 6), albeit once enrolled, none of them discontinued participation. Low participation may be attributable to family dynamics as children and parents may avoid expressing distressing emotions—particularly during acute treatment—in an effort to protect one other [47]. Moreover, especially for parents avoidance may play a key role in emotional adjustment [48] which may explain why some parents used the intervention for themselves but did not engage in involving their child as well.

Furthermore, the substantially lower FoP burden in children compared with parents [4, 9] could explain their lower participation. It is also worth noting that older children tend to report higher FoP than younger ones [9], and the underage participants of this study were, on average, in early adolescence (M_age_ = 13.27, SD = 3.15). At this developmental stage, children are better able to comprehend their illness and its potential progression and to reflect on future implications of their disease [13]. More broadly, age is one of the most common exclusion criteria in clinical trials involving children [45].

For future RCTs, concerns within families about sharing difficult feelings in the parent–child dyad, the potential benefits of strengthened family communication—including with younger children—should be addressed proactively to enhance participation and acceptability. Nevertheless, the absence of dropouts among enrolled minors suggests that KIPA may be suitable for adolescents; however, conclusions regarding younger children cannot be drawn from this sample. Notably, psychosocial professionals consider the intervention adaptable across different developmental stages [24].

### Efficacy

Since no evidence on the effectiveness of FoP interventions in paediatric oncology exists to date, and given marked differences in FoP between paediatric and adult oncology populations [7] this study provides preliminary results on the potential effects of KIPA; however, these findings should be interpreted cautiously due to the pilot nature of the study, the small sample size and the per-protocol analytic approach.

The group comparison (RQ 2a) provides preliminary results suggesting that parental FoP may be reduced with KIPA. The large postintervention effect may be overestimated due to the small sample size [49], yet it is noteworthy that this reduction in FoP was observed even though both highly and moderately burdened participants were included. Adult oncology interventions for patients and relatives to reduce FoP have small but robust effects [17]. Although the post hoc power analysis did not reveal the standard value of 1-ß=0.80, it was close and our findings may help informa future RCTs. Owing to the small sample size, we could not perform this analysis for children (control group, *n* = 1; intervention group, *n* = 5). The observed preliminary effects are consistent with the theoretical foundations of KIPA and suggest that processes such as reflecting on uncertainty, carefully approaching FoP-triggering situations, and strengthening individual and family resources might relate to more adaptive coping in parents. However, given the exploratory nature of this pilot study, such interpretations remain hypothetical.

Further secondary outcomes (RQ 2b) partly reflect known correlations of FoP with other psychosocial outcomes, such as HRQoL [5, 10] or PTSS [5]. The improvement in PTSS may reflect the narration of potentially traumatic events (e.g., the moment of diagnosis) of the cancer experience [50]. The reduction in anxiety may be related to psychoeducative elements of KIPA, which focus on anxiety-related coping behaviour [51]. In contrast, KIPA has no significant effect on depression, as behavioural activation—a main element used to treat depression [52]—is not part of KIPA. Likewise, improvements in the physical health subscale of HRQoL were smaller than those of the mental health subscale, which aligns with KIPA`s emphasis on strengthening psychosocial resources rather than physical functioning [53].

To answer RQ 2c, we analysed the longitudinal course of FoP. Before the intervention, FoP scores were similar to those reported in previous studies with parents [5–7, 9] and children [4, 8, 9]. We observed a pattern of decreasing parental FoP over time following the KIPA intervention, with high statistical power. Because the analyses for children`s FoP were underpowered, the pre–post effects of the intervention remain inconclusive. Despite non-significant post hoc findings, the longitudinal trend—particularly from BL to T2—indicate a possible decrease in children’s FoP, that may reflect an indirect benefit via parental modelling of coping with FoP-related triggers [13].

The pre–post analyses showed reductions in parental FoP from baseline to T1 in both AcT and FuC (RQ 2d). These preliminary within-group improvements, with effect sizes in the moderate-to-large range and high statistical power, suggest that improvements over time may occur across treatment phases which is particularly relevant given the enduring burden associated with FoP [7]. The findings also correspond with research showing lasting positive effects of family communication about FCR in AcT [15]. One might hypothesize that KIPA may be applicable across phases of the cancer trajectory, functioning in a more peritraumatic manner during acute treatment—when concerns primarily centre on uncertainty about treatment success—and shifting toward more post-traumatic processes in follow-up care, where fears predominantly relate to recurrence, potential consequences for the child’s development and late effects [24].

Sociodemographic characteristics (RQ 2e) such as parental education, perceived financial security, and the child’s age did not contribute significantly to explaining FoP levels at T1 when baseline FoP was taken into account. This pattern aligns with the assumption that baseline psychological burden is the primary determinant of parental FoP, while demographic factors play a comparatively minor role in short-term change. Nonetheless, the limited sample size and restricted variability in sociodemographic indicators constrain the strength of these conclusions.

### Limitations

A major limitation of this study is the small sample size, which is common in this area of care [54]. This limitation is particularly pronounced for the child subgroup, as only very few children participated. Consequently, the findings for children—especially younger children—must be interpreted with caution, and no firm conclusions can be drawn for this age group. Future studies will require substantially larger and more age-representative paediatric samples to adequately assess intervention effects in children.

As a result of COVID-19 pandemic-related constraints, the study faces several limitations. The suspension of randomisation—as described in the Methods section—reduced allocation randomness and may have introduced selection bias, potentially affecting baseline comparability between groups and limiting causal interpretation of treatment effects. Effect estimates should therefore be interpreted cautiously and confirmed in a fully randomised trial. Furthermore, the necessity to conduct a per-protocol analysis may bias treatment effects due to selective attrition. Although we conducted a dropout analysis, no meaningful or systematic differences between completers and non-completers emerged, suggesting that the influence of dropout on the findings is likely limited. Nevertheless, the results should be interpreted with caution and replicated in a fully powered RCT. In general, to mitigate such limitations in future research, alternative designs beyond waiting-list controls—such as parallel-group or cluster-randomised trials—should be considered for the acute setting.

Another limitation concerns the inclusion of non-indipendent observations within some families, as in three cases both parent took part. This may introduce within-family correlations and partially violate the assumption of independent observations. Given the small number of such dyads in this pilot trial, any resulting bias is expected to be minimal; nonetheless, this potential non-independence should be considered when interpreting the results. Future studies with larger and more diverse samples should employ analytic strategies that explicitly account for clustering at the family level.

### Conclusion and implications for psychosocial providers and future research

To sum up, the feasibility results of this study were mixed—particularly with regard to the participation of children, enrolment in follow-up care, and drop-out in acute treatment—and we identified key areas requiring optimisation before larger trials can be undertaken. Given the methodological limitations outlined above, the efficacy findings should be interpreted cautiously and regarded as preliminary. Nevertheless, the data provide indications on the positive effect of KIPA on parents of children with cancer, who are among the relatives most affected by FoP [7] and there are tentative signals of efficacy for adolescents. Additionally, the results give first hints of possible efficacy in different clinical settings.

To demonstrate the effectiveness of KIPA, especially in children, large multicentre trials are necessary—ideally incorporating reliable RCT designs suitable for acute care settings and appropriate analyses of dyadic associations. Future research should also examine how sociodemographic factors, including school attendance and the caregiving responsibilities of the participating parent, may influence KIPA’s outcomes. Additionally, further studies are warranted to assess the applicability and effectiveness of KIPA in other cohorts of children with chronic health conditions (e.g., cystic fibrosis) [55].

## Supplementary Information

Supplementary Material 1.

## Data Availability

Anonymised data were deposited at PsychArchives repository.
