# Supplementary material for: An intervention for fear of progression in childhood cancer patients and their parents: feasibility and preliminary efficacy in a pilot randomised controlled trial
Source: BMC Pediatr. 2026 Jul 13;26:652. doi: 10.1186/s12887-026-07201-x (PMC13362018; doi:10.1186/s12887-026-07201-x)
Supplement: Supplementary file 1 — Supplementary Material 1. [file 12887_2026_7201_MOESM1_ESM.docx]

Appendix 1

| Content of the KIPA modules (8 sessions à 60-90 minutes) | | |
| --- | --- | --- |
| Psycho- | Expanding knowledge about anxiety and FoP in particular by: | |
| education | • reflecting common anxiety effects and how they are experienced physically | |
| (family | • reflecting functional aspects of FoP and signs of dysfunctional FoP |  |
| dyad) | • reflecting existing ideas and different styles of coping with FoP | |
|  | Psychoeducation is supported by a comic with a main character (rabbit), who experiences various anxiety situations. Physical symptoms, typical reactions and how anxiety works are explained. FoP is described as a particular form of anxiety that is based on a real threat symbolised by a bear, which can be dangerous but there are also harmless types of bears (e.g. teddy bears, gummy bears). The threat is thus framed as one possibility among others, which has a certain probability of occurrence. The rabbit imagines the incounter with the most dangerous bear from a safe distance (through a telescope) and various coping strategies are presented. Ideas for individual coping strategies can be explored. The rabbit did not meet the dangerous bear, but sees the bears frightening shadows everywhere. This symbol can be used to explain the dysfunctional aspects of FoP. One person intervention: The perspecitve of other family members (e.g. the ill child) is asked using circular conversation techniques. | |
|  |  |  |
| Anxiety- | Exploring fears and worries about the further course of the child's cancer by: | |
| confron- | • naming specific situations in which FoP is perceived | |
| tation | • analyzing triggers, behavior and course of the perceived FoP | |
| (individual) | Clients are asked to look through an imaginary telescope and name specific situations in which they had illness-related fears. Triggers, duration and reactions are described and assessed in terms of functionality, with short-term fears and adaptive coping strategies in the case of a specific threat being considered as functional. | |
| Resource- | Activating resources by: | |
| activation | • drawing a biographical timeline/ the course of the disease | |
| (individual) | • reflecting helpful things in crisis or positive lifetime periods | |
|  | • compiling helpful coping skills | |
|  | In order to expand coping strategies in dealing with FoP, an exploration and compilation of existing skills and knowledge along biographical experiences (e.g. the illness) now takes place. | |
| Training | Creating a family shared perspective and applicating coping skills by: | |
| (family | • sharing FoP situations and helpful coping skills in the family dyad | |
| dyad) | • training concrete situations the family has probably to cope with | |
|  | The anxiety situations experienced and the resources explored are shared in the family dyad using the reflecting team method. The family's handling of specific anxiety situations is trained (e.g. by role play). One person intervention: Anxiety situations and resources of other family members (e.g. the ill child) are explored using circular conversation techniques and presented by the therapist. | |

Appendix 2

Regression Model Predictors of FoP scores at T1

Table 1: Multiple linear regression predicting FoP scores at T1 from baseline FoP, child’s age, perceived level of financial security (LFS), and educational level (standardized coefficients).

|  | Estimate (ß) | Std. Error | T value | p | 95%-KI (LL) | 95%-KI (UL) |
| --- | --- | --- | --- | --- | --- | --- |
| (Intercept) | –1.272 | 7.144 | –0.178 | .860 | -0.36 | 0.22 |
| Baseline FoP_z | 0.771 | 0.164 | 4.701 | <.001 | 0.43 | 1.07 |
| Age of the child_z | -0,08005 | 0.14424 | -0.555 | 0.584 | -0.38 | 0.22 |
| LFS_z | -0.20597 | 0.14414 | -1.429 | 0.166 | -0.50 | 0.09 |
| Education_z | -0.13055 | 0.15344 | -0.851 | 0.403 | -0.45 | 0.19 |

Notes: β = standardized regression coefficients. SE = standard error. CI = confidence interval, LL = lower limit, UL = upper limit.

Model fit: R² = .52 (adjusted R² = .44), F(4, 24) = 6.40, p = .001.
The model was statistically significant and explained 52% of the variance in T1 FoP scores.

As expected, the baseline FoP score was highly significant and predicted the T1 FoP value with a large effect size. The remaining predictors—child’s age, perceived level of financial security (LFS; lower scores indicate better financial security), and educational level—did not show statistically significant effects. However, their 95% confidence intervals indicate that small to moderate effects cannot be ruled out so that individuals with poorer financial security and lower education may tend to benefit less from the intervention.

Multicollinearity: All VIF values were unproblematic and ranged between 1.03 and 1.19.

Heteroscedasticity: The Breusch–Pagan test indicated no evidence of heteroscedasticity

(BP = 2.62, p = .623), which aligned with visually acceptable residual plots.

Residual distribution: The QQ-plot indicated an approximately normal distribution, with minor deviations typical for small samples.

Influence diagnostics identified one potentially influential observation (Case 11), with a high DFBETA for LFS (≈ 1.0) and an elevated Cook’s distance (0.23). After re-estimating the model without this case, results remained largely unchanged:

- Baseline FoP remained significant and strong (β = 0.76, p < .001).
- LFS (β = –0.06, p = .723) and Education (β = –0.21, p = .207) remained nonsignificant.
- Model fit was nearly identical (R² ≈ .53, adjusted R² = .45, F(4, 24) = 6.53, p = .001).

The sensitivity analysis showed that the small negative effect of perceived financial security (LFS) was largely driven by a single influential case and disappeared once this case was removed. In contrast, the effect of education became slightly stronger after excluding the case, suggesting a suppressed underlying trend, though it remained non‑significant. Overall, neither LFS nor education demonstrated a stable or reliable influence on intervention outcomes.
